# RGB-D SLAM in Low-Texture Indoor Environments: Complementary Structural Constraints and Resource-Aware Mapping

**DOI:** 10.3390/s26216866

**Published:** 2026-10-30

**Authors:** Fei Nie, Zhuohang Chen, Zhuoling Xiao, Xinchen Zhang, Shujie Li

**Affiliations:** School of Information and Communication Engineering, University of Electronic Science and Technology of China, Chengdu 611731, China; 202312012428@std.uestc.edu.cn (F.N.); 2023190503030@std.uestc.edu.cn (Z.C.); 202221011616@std.uestc.edu.cn (X.Z.); 202522011435@std.uestc.edu.cn (S.L.)

**Keywords:** RGB-D sensing, visual SLAM, sensor data fusion, low-texture indoor scenes, point–line–plane features, structural mapping, visual place recognition

## Abstract

Low-texture indoor scenes weaken repeatable point correspondences while often retaining geometric information in image boundaries and depth-supported surfaces. We examine the pose-estimation benefits and resource costs of combining these complementary measurements in RGB-D SLAM. The evaluated system integrates point-reprojection, endpoint-to-line, direct-plane, and structural plane-relation residuals using information scales and modality coefficients fixed before evaluation. Multiple observations of the same physical plane from frames selected for map insertion are consolidated into persistent plane instances, while a compact indoor visual vocabulary supports keyframe-level place recognition. On TUM RGB-D fr3/structure_notexture_near, translational ATE RMSE decreased from 0.052350 m for point only (P) to 0.021353 m for point–line (P + L) and 0.017041 m for point–line–plane (P + L + Π, where P denotes point, L line, and Π plane). Median tracking-call latency increased from 16.174 to 50.589 and 92.505 ms, respectively. The P + L + Π ATE RMSE was 10.3–18.9% below the published results of three closely related PLP methods on the same sequence. Across four ICL-NUIM sequences, reconstruction RMS remained within 0.020–0.055 m. The indoor vocabulary reduced storage from 145.3 to 15.7 MB and loading time from 3235 to 255 ms. Across three P + L + Π runs and 221 valid resource samples, median resident set size was 840.5 MiB, peak resident set size remained below 1.08 GiB, median CPU demand was approximately 1.84 logical cores, and swap consumption remained zero. The results quantify the trajectory-accuracy gains and computational costs of complementary geometric measurements in low-texture indoor RGB-D SLAM.

## 1. Introduction

White walls, long corridors, and repetitive layouts are common in indoor environments yet remain challenging for visual SLAM. Their weakly textured or repetitive appearance yields few distinctive feature point correspondences, which can make point-based tracking unreliable. A registered RGB-D frame, however, provides more than point texture: intensity edges align with wall intersections and door frames, while the organized depth map captures extended planar surfaces such as walls and floors. Low image texture thus degrades point-based measurements without necessarily eliminating these complementary geometric cues. This observation motivates the use of geometric constraints alongside point features in texture-poor indoor scenes.

ORB-SLAM2 and ORB-SLAM3 provide accurate and efficient tracking when repeatable point features are present [1,2]. Hybrid SLAM systems augment point measurements with complementary geometric primitives. PL-SLAM adds line correspondences [3]. PlanarSLAM and ManhattanSLAM exploit plane observations and structural regularities [4,5]. SP-SLAM combines points with surfels [6]. Point–line–plane systems further integrate heterogeneous features for localization and mapping [7,8,9]. Recent surveys identify low texture and the balance among robustness, accuracy, and computation as central RGB-D SLAM concerns [10]. Evaluating such systems, however, requires more than trajectory error alone: a complete evaluation must also account for tracking cost and for the plane-instance map and visual-word database maintained across frames.

The plane-instance map and visual-word database serve two distinct functions in the evaluated system. Geometrically, multiple observations of the same physical plane from frames selected for map insertion are associated and fused into plane instances that accumulate structural support across views. Visually, keyframe descriptors are quantized with a compact indoor vocabulary for place recognition. The plane map and the visual-word database therefore preserve different information, but both connect frame-level tracking to longer-term operation.

The associated costs arise during per-frame tracking, keyframe-level map updates, initialization, and whole-process operation. Feature extraction and pose optimization affect per-frame or per-tracking-call latency; fusion of plane observations from frames selected by the keyframe-insertion policy (Section 3.1) determines how plane instances persist and accumulate; vocabulary storage and loading define model and initialization costs; and sampled resident memory and CPU utilization describe whole-process demand.

This paper examines structured indoor scenes in which point support becomes weak while depth planes and long intensity edges at wall intersections and door frames remain observable. The tracker extracts fixed AGAST 5–8 corners [11], EDLines segments [12], and organized-depth planes [13]. Point-reprojection, endpoint-to-line, direct-plane, and structural plane-relation residuals jointly constrain the current camera pose $T_{cw}$, which maps world coordinates into the current camera frame. Their information scales remain fixed, and the P + L + Π modality coefficients remain constant within each run. A common reference configuration is used across sequences, and the sensitivity analysis compares predefined fixed-weight configurations. Effective measurement contributions can still vary with scene-dependent association, robust weighting, and outlier rejection because the active observations and retained inliers change from frame to frame.

The evaluation addresses geometric performance and resource use through complementary measurements. Configuration comparisons on a selected low-texture TUM RGB-D sequence examine trajectory accuracy together with tracking-call latency [14]. Four ICL-NUIM living-room trajectories characterize pose and surface-reconstruction performance [15]. Detector and vocabulary measurements quantify component costs, while process monitoring records sampled memory and CPU utilization. A physical corridor demonstration separately shows integrated structural mapping on a mobile platform.

The contributions are as follows:

- Complementary structural estimation under point-feature degradation. We integrate point-reprojection, endpoint-to-line, direct-plane, and structural plane-relation residuals within a robust RGB-D pose-estimation system for low-texture indoor scenes. Point reprojections and projected line endpoints constrain image alignment, direct-plane observations constrain surface orientation and distance, and parallel/perpendicular plane relations add orientation constraints. All four residual groups share the current camera pose in a robust optimization objective. The front end uses AGAST and EDLines, and the evaluated configurations are P, P + L, and P + L + Π. The configuration uses fixed nominal information scales and modality coefficients, while effective measurement contributions vary through scene-dependent association, robust weighting, and outlier rejection.
- Persistent structural and visual representations. Building on the plane-instance mapping strategy of PlanarSLAM [4], we associate and fuse multiple observations of the same physical plane from frames selected for map insertion into persistent plane instances for surface reconstruction. In parallel, a compact indoor visual vocabulary supports keyframe-level place recognition with lower model storage and loading costs than the evaluated general vocabulary. Together, these components maintain persistent geometric and visual representations alongside frame-level pose estimation.
- Explicit accuracy–resource characterization. We report configuration-level differences in trajectory accuracy and tracking latency, together with surface reconstruction error, detector timing, sampled process memory and CPU utilization, and vocabulary storage, loading, and query costs.

The remainder of this paper is organized as follows. Section 2 positions the system among point–line–plane estimation, structural mapping, and visual place recognition. Section 3 describes the measurement models and persistent representations. Section 4 defines the evaluation protocols, Section 5 reports the results, and Section 6 and Section 7 discuss their implications and summarize this study.

## 2. Related Work

Point features remain an effective default because binary descriptions support fast association and sparse bundle adjustment [1,2,16]. Their repeatability falls on uniform walls, under blur, or in repetitive layouts. PL-SLAM incorporates line segments into monocular tracking [3]. RGB-D point–line systems have been evaluated specifically in low-texture indoor scenes [17,18]. Recent sensors work also uses integrated point–line maps for visually weak structure [19]. Lines provide extended orientation and boundary information, but they also introduce endpoint-depth and association errors.

Recent point–line–plane systems have advanced heterogeneous geometric fusion. Structure PLP-SLAM supports monocular, stereo, and RGB-D inputs within a sparse formulation [7]. UPLP-SLAM unifies point, line, and plane representation, association, and joint optimization [20].

PLPF-VSLAM selects among point, point–line, point–plane, and point–line–plane modes according to feature availability [8]. The weak-texture PLP-SLAM of Wang et al. adds degeneracy-aware constraints and Manhattan-based pose initialization [9]. PLPM-SLAM further combines point–line–plane optimization with orthogonal-plane, virtual-plane, and heterogeneous-feature relations [21]. RGB-D SLAM with structural regularities and ManhattanSLAM add plane or layout relations [4,5]; SP-SLAM couples points with surfels [6], while point–plane systems use depth surfaces to constrain indoor pose [22]. Together, these systems establish complementary structural observations across distinct estimator configurations and evaluation protocols.

Dense RGB-D methods represent geometry using volumetric fusion, direct image alignment, or registered point clouds [23,24,25]. Modern dense representations can deliver high-fidelity rendering and mapping but require different computational and memory budgets [26]. In indoor scenes dominated by walls and floors, planar descriptions offer a complementary representation; planar semantic SLAM has also examined bandwidth and computation limits [27]. Our map retains point landmarks for tracking, associates observations of the same physical plane across keyframes with a persistent plane instance, and triangulates the fused points for visualization [28]. Multistage map views show how the persistent planes accumulate support, and reconstruction RMS measures the final surface error.

Loop-candidate retrieval uses hierarchical bags of binary words [29]. A vocabulary tuned to the deployment domain can be substantially smaller than a broad general vocabulary. Its model size, loading, and query costs are measured separately from tracking latency.

This study combines AGAST point extraction, EDLines segments, and the four residual families in a robust RGB-D pose estimator for low-texture indoor scenes. The residual formulation and plane-instance association build on PlanarSLAM, while hierarchical visual-word retrieval follows DBoW2. The system pairs these persistent geometric and visual representations with measurements of low-texture accuracy, latency, reconstruction, vocabulary cost, and whole-process demand.

## 3. Materials and Methods

### 3.1. System Overview and Sensor Assumptions

The input at time *t* is a registered pair $\left(I_{t},D_{t}\right)$ with fixed intrinsic calibration *K* and RGB–depth extrinsics. The method assumes the pair is synchronized by the dataset or acquisition pipeline. Points and lines are extracted from $I_{t}$; valid depth pixels are back-projected to an organized cloud from which planes are segmented. All three observation families constrain a single camera pose $T_{cw}\in \mathrm{SE}\left(3\right)$, which maps world coordinates into the current camera frame.

#### Keyframe-Insertion Policy

The keyframe-insertion policy follows the RGB-D policy of ORB-SLAM2 as used in the PlanarSLAM tracking framework [1,4]. After the post-relocalization interval, the conventional branch combines a minimum inlier count with the maximum-frame interval, an idle-mapper interval, or a weak-tracking condition. A separate structural trigger requests a keyframe when a newly observed plane has no map association. When local bundle adjustment is active, new keyframes can only be inserted if the pending keyframe queue remains shorter than a threshold of three keyframes. Frames inserted under this policy are termed accepted keyframes.

**Figure 1:**
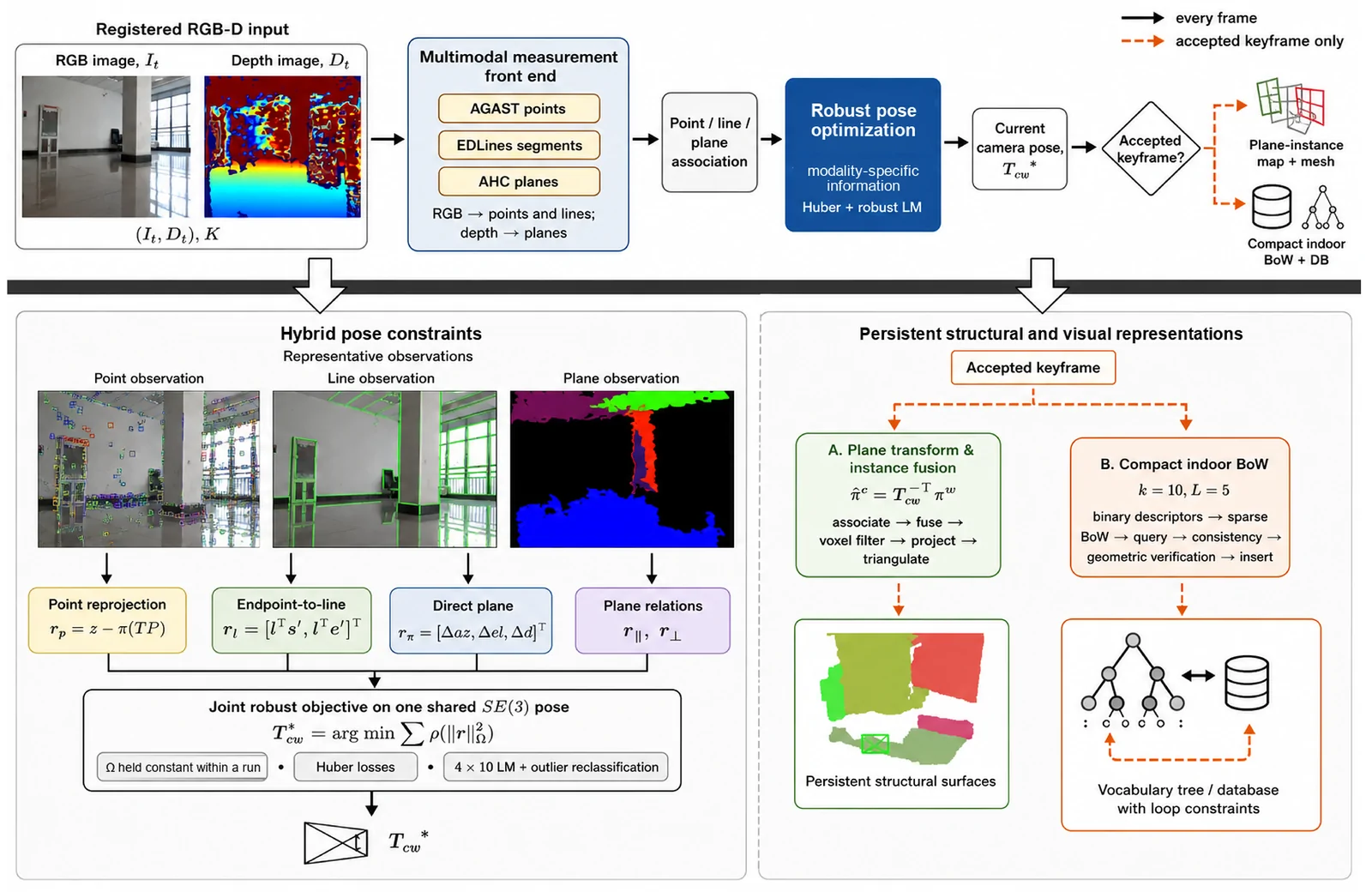
RGB-D sensing, tracking, mapping, and place-recognition pipeline. A registered RGB image supplies point and line observations, and its synchronized organized-depth image supplies planes. Solid arrows mark the every-frame path to fixed-information pose optimization. Dashed arrows mark accepted-keyframe operations: plane-instance fusion, meshing, descriptor quantization, database query and insertion, and geometric checking of loop candidates. The lower panel expands the point, line, direct-plane, and plane-relation residual groups and separates current-pose estimation from persistent representation updates. Black solid arrows indicate every-frame processing, whereas orange dashed arrows indicate accepted-keyframe operations. Yellow, green, blue, and purple residual blocks denote point, line, direct-plane, and plane-relation constraints, respectively. Colors in the observation and map examples distinguish displayed features or plane regions. The superscript * in $T_{cw}^{*}$ denotes the optimized camera pose.

Figure 1 separates two schedules. Extraction, association, pose optimization, and tracking-state updates occur for every RGB-D pair. Each accepted keyframe selected by the policy above triggers plane fusion, surface reconstruction, visual-word database insertion, and loop-candidate processing. Table 1 summarizes these schedules. Structural observations are extracted from every frame, whereas plane fusion and visual-word database updates are triggered by keyframe insertion.

### 3.2. Fixed AGAST Point Front End

AGAST organizes the accelerated segment test as an efficient decision tree [11]. The implementation fixes the eight-pixel 5–8 mask: a candidate is accepted when the learned decision sequence establishes a contiguous bright or dark segment under the detector threshold. The proposed system adopts this configuration as its point front end. Point candidates are extracted at multiple image-pyramid levels and distributed spatially within each level. An intensity-centroid orientation steers the 256-bit binary descriptor [16].

For a map point $P_{i}\in R^{3}$, observed image coordinate $z_{i}={\left[u_{i},v_{i}\right]}^{T}$, and calibrated projection π(·), the point residual is

(1) $$r_{i}^{p}=z_{i}-\pi \left(T_{cw}P_{i}\right).$$

The information matrix $\Omega_{i}^{p}$ is the inverse variance associated with the fixed image-pyramid level. The point extractor therefore determines the residual scale from the pyramid level.

### 3.3. Line Observations and Residual

EDLines returns extended intensity segments and controls false detections through the edge-drawing formulation [12]. The implementation derives its gradient threshold from the current image distribution, and descriptor association combines binary appearance and geometric consistency. For homogeneous segment endpoints ${\bar{s}}_{j}$ and ${\bar{e}}_{j}$, the observed image line is implemented as

(2) $$l_{j}=\frac{{\bar{s}}_{j}\times {\bar{e}}_{j}}{∥{\bar{s}}_{j}\times {\bar{e}}_{j}{∥}_{2}},\;{∥l_{j}∥}_{2}=1.$$

Let $\left(S_{j},E_{j}\right)$ be the endpoints of the matched persistent 3D map line. Their projected homogeneous image coordinates are ${\bar{s}}_{j}^{\prime }$ and ${\bar{e}}_{j}^{\prime }$. The endpoint-to-line residual is

(3) $$r_{j}^{l}={\left[\begin{array}{cc} l_{j}^{T}{\bar{s}}_{j}^{\prime } & l_{j}^{T}{\bar{e}}_{j}^{\prime } \end{array}\right]}^{T},\;s_{j}^{\prime }=\pi \left(T_{cw}S_{j}\right),\;e_{j}^{\prime }=\pi \left(T_{cw}E_{j}\right).$$

Both endpoints must agree with the observed infinite line. Equation (2) normalizes the complete homogeneous line vector, so Equation (3) defines an algebraic homogeneous endpoint-to-line residual. Unit normalization of the line normal $\left(a_{j},b_{j}\right)$ defines the corresponding Euclidean endpoint-to-line distance in pixels. The two scalars are instantiated as separate unary edges. Each edge stores the scalar in the first entry of a three-vector whose other entries are zero and uses $I_{3}$, equivalent to unit weight on the active scalar. Robust estimation follows the fixed implementation settings summarized in Table 2: a Huber loss limits the influence of large endpoint residuals, and the complete segment is rejected when either endpoint violates the prescribed chi-square residual threshold.

### 3.4. Organized-Depth Planes and Residual

Depth samples first undergo the configured validity checks before plane extraction. The resulting cloud preserves image organization and is partitioned on a 10×10 grid for agglomerative hierarchical plane extraction [13]. The TUM configuration requires 2000 support pixels and a 0.05 m extraction distance, whereas the ICL configuration uses 500 pixels and 0.03 m. Each plane is stored in Hesse form $\pi ={\left[n^{T},d\right]}^{T}$ with ${∥n∥}_{2}=1$. Coefficients are normalized and the complete four-vector is sign-flipped when its fourth coefficient is negative, making the sign deterministic. Candidate association uses a 0.05 m plane-distance threshold and a normal-dot-product threshold of 0.985. For normalized plane normals $n_{a}$ and $n_{b}$, a parallel relation requires $|n_{a}^{T}n_{b}|>\tau_{\|}$, and a perpendicular relation requires $|n_{a}^{T}n_{b}|<\tau_{⊥}$. We set $\tau_{\|}=0.9962$ and $\tau_{⊥}=0.08716$, corresponding to approximately $5^{\circ }$ from parallel alignment and an angle between $85^{\circ }$ and $95^{\circ }$, respectively. Both thresholds remain fixed throughout a run.

For a world plane $\pi_{k}^{w}$, the predicted camera-frame plane is

(4) $${\hat{\pi }}_{k}^{c}=T_{cw}^{-T}\pi_{k}^{w}.$$

Let $\hat{n}$ and $\tilde{n}$ be the normalized predicted and observed normals. We define $R(\hat{n})$ as the azimuth–elevation rotation associated with $\hat{n}$. The implementation first expresses the observed normal in a chart local to the prediction, $n_{\mathrm{rel}}=R{\left(\hat{n}\right)}^{T}\tilde{n}$, and then uses

(5) $$r_{k}^{\pi }=\left[\begin{array}{c} atan2(n_{\mathrm{rel},y},n_{\mathrm{rel},x})\\ atan2\;\left(n_{\mathrm{rel},z},\sqrt{n_{\mathrm{rel},x}^{2}+n_{\mathrm{rel},y}^{2}}\right)\\ \hat{d}-\tilde{d} \end{array}\right].$$

The angular components constrain camera orientation relative to dominant surfaces, while the distance component constrains translation along their normals. The implementation uses numerical finite-difference Jacobians.

Matched structural relations add two orientation-only plane edges. For a predicted plane normal $\hat{n}$ and observed normal $\tilde{n}$, let $\psi_{\|}\left(\hat{n},\tilde{n}\right)$ sign-align $\hat{n}$, rotate it to the local *x* axis, and return the azimuth and elevation of $\tilde{n}$ in that chart. Let $\psi_{⊥}\left(\hat{n},\tilde{n}\right)$ first rotate $\hat{n}$ by π/2 about the normalized cross-product axis and then apply the same chart. The active relation residuals are   

(6) $$r_{q}^{\|}=\psi_{\|}\left({\hat{n}}_{q},{\tilde{n}}_{q}\right),\;r_{h}^{⊥}=\psi_{⊥}\left({\hat{n}}_{h},{\tilde{n}}_{h}\right),$$

both in $R^{2}$. These relation edges constrain the dominant parallel and orthogonal orientations.

### 3.5. Fixed-Information Robust Pose Optimization

The residual groups share the pose variable and retain modality-specific fixed information matrices. Pose estimation solves

(7) $$\begin{array}{cc} T_{cw}^{*}=\arg\min_{T_{cw}}\; & \sum_{i\in P}\rho_{p}\;\left({\left(r_{i}^{p}\right)}^{T}\Omega_{i}^{p}r_{i}^{p}\right)+\sum_{j\in L}\sum_{m\in \{S,E\}}\rho_{l}\;\left({\left(r_{jm}^{l}\right)}^{2}\right)\\ \; & +\sum_{k\in S}\rho_{\pi }\;\left({\left(r_{k}^{\pi }\right)}^{T}\Omega_{k}^{\pi }r_{k}^{\pi }\right)\\ \; & +\sum_{q\in R_{\|}}\rho_{\|}\;\left({\left(r_{q}^{\|}\right)}^{T}\Omega^{\|}r_{q}^{\|}\right)+\sum_{h\in R_{⊥}}\rho_{⊥}\;\left({\left(r_{h}^{⊥}\right)}^{T}\Omega^{⊥}r_{h}^{⊥}\right), \end{array}$$

where all ρ terms are Huber losses [30]. Point information follows the fixed pyramid variance, and line information is unit scale on each active algebraic endpoint residual. Direct-plane information combines fixed angular and distance entries, while each relation edge uses a fixed two-dimensional angular scale. The optimizer keeps these relative scales fixed as detected-feature counts, match counts, inlier fractions, and residual distributions vary. Levenberg–Marquardt runs four robust rounds of ten iterations, with fixed residual thresholds reclassifying outliers between rounds. Table 2 summarizes the optimizer settings.

The P + L + Π reference configuration uses point/line/plane weights of 0.70/0.15/0.15. This gives point observations a larger nominal share while retaining complementary line and plane constraints. Equal line and plane weights provide a symmetric initial structural configuration and reduce the number of independently adjusted coefficients. The same reference setting is used across evaluation sequences for consistent comparison and reproducibility. In the sensitivity analysis, the coefficients vary between predefined configurations but remain fixed within each run. Thus, the selected configuration contains no per-frame adaptive change in modality weights. Nominal coefficients do not determine fixed contribution fractions: actual influence also depends on the number of active edges, residual dimensions and scales, information matrices, Huber weights, and outlier rejection.

Per-frame processing first acquires registered RGB-D input and extracts and associates points, lines, and planes. The system then constructs the residual sets, performs robust pose optimization and outlier reclassification, and updates tracking. Accepted keyframes additionally trigger map-entity fusion and place-recognition updates.

### 3.6. Persistent Plane Instances and Surface Construction

Each accepted keyframe plane is transformed to the world frame and compared with persistent instances by fixed normal, distance, and visibility tests. A matched observation contributes its support points to the existing instance; otherwise, a new plane instance is created. Fused support is voxel filtered, projected to the plane support, indexed spatially, and passed to greedy triangulation [28]. The resulting mesh represents dominant structural surfaces while preserving plane identity. Small gaps supported by neighboring planar regions are filled after triangulation. We evaluate plane-instance consolidation through its progression across keyframes and the final reconstruction error. The corridor experiment separately tests the same mapping path on the robot platform.

### 3.7. Compact Indoor Bag of Words

Place recognition follows DBoW2’s hierarchical binary-word formulation [29]. The indoor vocabulary was trained from 4733 RGB images from TUM RGB-D, ICL-NUIM, and additional indoor captures. Vocabulary training used sequences disjoint from the TUM and ICL-NUIM evaluation sets, with every evaluation frame held out. The indoor vocabulary uses a branching factor k=10 and depth L=5, compared with k=10 and L=6 for the general ORB vocabulary. At each nonleaf node, binary descriptors are assigned to a child cluster; a leaf represents a visual word. Keyframe descriptors are quantized into a sparse word vector, scored against the keyframe database, and passed to covisibility consistency and geometric verification before a loop constraint is accepted. The database is queried and then updated only at keyframes.

The reduced tree lowers storage, loading, and query costs while changing visual-word resolution. These resource costs are evaluated separately in Section 5.

## 4. Experimental Setup

### 4.1. Evaluation Questions and Protocol Overview

The evaluation follows the three contributions stated in Section 1. First, does complementary point–line–plane estimation improve pose accuracy and tracking availability when point support is weak? Second, how are the persistent plane and visual representations characterized through structural reconstruction and place-recognition costs? Third, what accuracy–latency behavior and resource demand are observed across the evaluated configurations and complete P + L + Π process? Table 3 links these questions to the corresponding datasets, measurements, and system levels.

### 4.2. Protocol for Complementary Structural Estimation

Tracking was evaluated on four structured sequences from TUM RGB-D [14]. The sequence labels are notexture_far, notexture_near, texture_far, and texture_near, with durations of 27.28, 36.44, 31.55, and 36.92 s, respectively. The modality ablation and PLP comparison use rgbd_dataset_freiburg3_structure_notexture_near. The point-only baselines were ORB-SLAM2 [1] and ORB-SLAM3 [2]. Published PLP results were obtained for PLPF-VSLAM [8], PLP-SLAM [9], and UPLP-SLAM [20].

The TUM evaluation reports translational ATE RMSE in meters after rigid SE(3) alignment with fixed scale. Estimated and ground-truth poses are associated within 0.02 s. Translational RPE is reported in meters per step and rotational RPE in degrees per step, where one step connects adjacent associated poses. Tracking-call latency is reported separately in milliseconds. Only trajectories with a sufficient number of successfully associated poses are considered valid for these calculations.

The competing PLP values are published ATE RMSE results on the same TUM sequence. This sequence-matched comparison directly uses the values reported by the respective studies.

### 4.3. Protocol for Persistent Structural and Visual Representations

Four ICL-NUIM living-room trajectories, lr_kt0–3, supplied the synthetic pose and reconstruction benchmarks [15]; they contain 1510, 967, 882, and 1242 frames. Pose error is evaluated using the unitless RMSE of the full-transformation error after full-trajectory Sim(3) Umeyama alignment. Surface reconstruction error is reported separately as RMS in meters, and mapping progression is inspected across accepted keyframes.

The lr_kt1 visualization compares the proposed method, ORB-SLAM3, ElasticFusion, and InfiniTAM. The ElasticFusion and InfiniTAM positions are taken from public SLAMBench run logs [31]. After conversion to the official ICL-NUIM coordinate convention, positions are associated with livingRoom1.gt.freiburg by frame index. Each estimated trajectory is independently aligned to ground truth on the 965 common valid frames using full-trajectory Umeyama Sim(3) alignment. Absolute position error (APE) is the Euclidean distance between each aligned position and its ground-truth counterpart, reported in meters.

The compact indoor vocabulary is compared with the evaluated general ORB vocabulary using model storage, loading time, and one-query latency. These measurements characterize the persistent visual representation; mapping progression and reconstruction RMS characterize the structural representation.

The onboard prototype used an Intel RealSense D435i RGB-D camera (Intel Corporation, Santa Clara, CA, USA) and an NVIDIA Jetson Xavier NX with 8 GB memory (NVIDIA Corporation, Santa Clara, CA, USA). RGB-D acquisition used a resolution of 640×480, with the camera configured at 15 frames/s. The experiment used RGB-D sensing alone. The platform traversed an indoor corridor of approximately 40 m and produced a plane map.

### 4.4. Protocol for Accuracy–Resource Characterization

The desktop software environment used Ubuntu 18.04 (Canonical Ltd., London, UK), Oracle VirtualBox 7.0 (Oracle Corporation, Austin, TX, USA), and Windows 11 (Microsoft Corporation, Redmond, WA, USA). The implementation used OpenCV 3.4.1, PCL 1.9.0, Eigen 3.1.0, and g2o revision 67cbe15c998737ac6705d3cd18201a72be0d073d. The locally modified PlanarSLAM and DBoW2 implementations were versions 2.0.1 and 1.0, respectively.

The detector-only benchmark was executed in an Ubuntu 18.04 VirtualBox 7.0 guest configured with 10 vCPUs and 8 GB RAM. The virtual machine ran on a Windows 11 host equipped with an Intel Core i7-12700K processor and 16 GB physical memory. The benchmark compares five segment-test masks on the same four TUM sequences and reports mean detector call time and returned corners per frame. These measurements characterize front-end speed and candidate density under common hardware and inputs.

Process resources were profiled in the Ubuntu 18.04 VirtualBox 7.0 guest configured with 10 vCPUs and 8 GB RAM on the Windows 11 i7-12700K host. Three independent P + L + Π executions were monitored at a median sampling interval of 2 s. A sample was retained when resident set size (RSS), SLAM-process CPU, and system CPU were all numeric. This rule yielded 79, 77, and 65 samples from the three runs, respectively, and 221 samples in total. RSS is the physical memory resident for the monitored process and is reported in MiB. Under the Linux convention used by the monitor, 100% process CPU corresponds approximately to one logical core. System CPU reports host-wide utilization. Swap consumption was calculated from total minus free swap. The pooled distributions are summarized by their median, 95th percentile, and maximum.

### 4.5. Fixed-Weight Sensitivity Protocol

We evaluated five point/line/plane configurations: 0.34/0.33/0.33, 0.60/0.10/0.30, 0.60/0.30/0.10, the reference 0.70/0.15/0.15, and 0.80/0.10/0.10. Each configuration was run three times on each of the four TUM sequences and ICL-NUIM lr_kt0–3, yielding 120 runs in a balanced comparison. Adaptive weighting was disabled throughout. The remaining code and configuration were unchanged between the reference and alternative settings.

All sensitivity trajectories use fixed-scale SE(3) alignment. TUM uses the 0.02 s association tolerance above; ICL uses a one-frame association offset: the ground-truth frame identifier equals the estimated frame identifier plus one. We report translational ATE RMSE, adjacent-associated-pose translational and rotational RPE RMSE, associated-pose counts and coverage (associated poses divided by processed frames), and tracking-call latency. Group summaries use the median and [minimum, maximum] of three runs. These translational metrics are separate from the unitless full-transformation Sim(3) index used for the cross-trajectory pose comparison.

## 5. Results

### 5.1. Complementary Constraints Under Point-Feature Degradation

**Figure 2:**
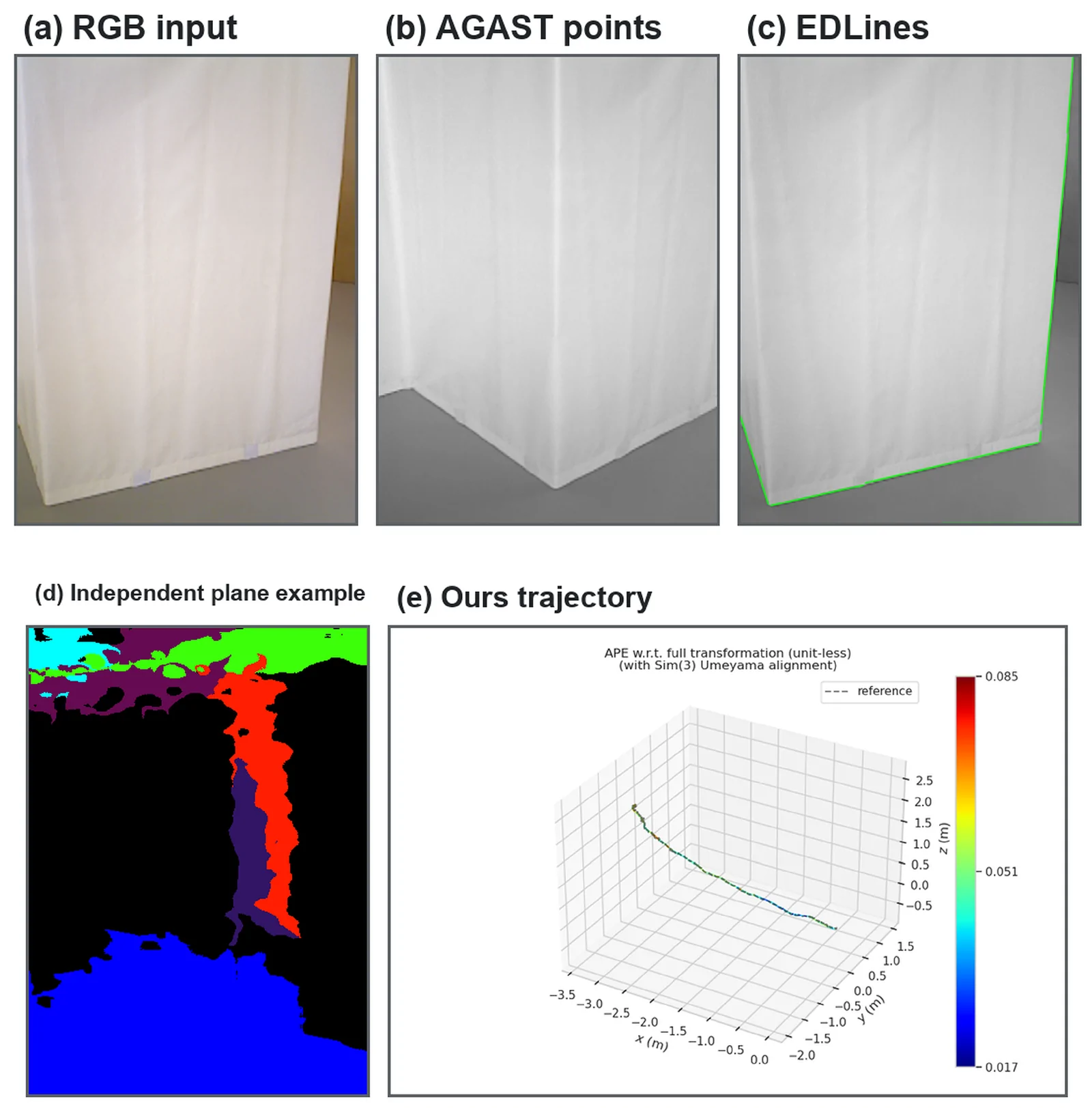
Observation evidence in the target regime. (**a**) A TUM notexture_far RGB frame. (**b**) AGAST yields almost no point support in the same frame. (**c**) EDLines retains extended wall and frame boundaries. (**d**) Organized depth yields large plane regions in a separate corridor example. (**e**) An aligned trajectory visualization for notexture_far using a full-transformation APE color scale. Quantitative TUM results follow the fixed-scale SE(3) protocol in Section 4. Together, the panels illustrate complementary observation and trajectory evidence under scarce point support. In (**c**), green segments indicate detected line features. In (**d**), colors distinguish extracted plane regions against the black background. In (**e**), the trajectory color indicates the unitless full-transformation APE, increasing from blue to red as shown by the color bar; the dashed line is the reference trajectory.

Figure 2 shows the observations available when point texture degenerates. Panels (a)–(c) compare point and line extraction on the same TUM frame; panel (d) shows organized-depth plane extraction in a separate corridor scene, and panel (e) presents the aligned trajectory visualization for notexture_far.

Table 4 compares the predefined P, P + L, and P + L + Π configurations under the same trajectory protocol. Their observed translational ATE RMSE values were 0.052350, 0.021353, and 0.017041 m, respectively. The P + L value was 59.2% below P, while P + L + Π was 20.2% below P + L and 67.4% below P. The same ordering appears in one-step RPE: translational RPE decreased from 0.016474 to 0.008259 and 0.007064 m/step, while rotational RPE decreased from 0.905 to 0.512 and 0.447 deg/step.

The P + L + Π configuration produced 1054 estimated poses, of which 1052 were associated with ground truth, and tracking remained available throughout the sequence. Median and 95th-percentile translational ATE were 0.014341 and 0.029194 m.

Each frame contained a median of 857 points, 16 lines, and 3 planes. Median feature-extraction, internal-tracking, and frame-total times were 47.563, 41.417, and 91.651 ms, respectively. Median tracking-call latency was 92.505 ms.

Table 5 reports trajectory availability on the two no-texture sequences. ORB-SLAM2 and ORB-SLAM3 produced zero valid trajectories across ten runs on each sequence. The proposed system produced an evaluable trajectory on both sequences, associating complementary line and plane measurements with trajectory availability under scarce point support.

Table 6 compares results on fr3/structure_notexture_near. The proposed P + L + Π configuration achieved an ATE RMSE of 0.0170 m, corresponding to reductions of 10.3%, 14.8%, and 18.9% relative to the published results of PLPF-VSLAM, PLP-SLAM, and UPLP-SLAM, respectively. These percentages use the unrounded value of 0.017041 m reported in Table 4; 0.0170 m is the display precision used for the literature comparison.

### 5.2. Evaluation of Persistent Structural and Visual Representations

The ICL-NUIM pose comparison first characterizes the trajectories used by the structural mapping evaluation. The subsequent mapping and vocabulary results then describe the persistent geometric and visual representations maintained beyond the current frame.

Table 7 shows that the lowest-error method varies by sequence. SP-SLAM has the lowest values on lr_kt0 and lr_kt2, ManhattanSLAM on lr_kt3, and the proposed method on lr_kt1. The proposed values span 0.019523–0.026922 across the four trajectories. These methods use different geometric representations: SP-SLAM combines points and surfels, ManhattanSLAM exploits Manhattan-frame structure, and the proposed estimator combines point, line, direct-plane, and plane-relation residuals. Their sequence-specific rankings should therefore be interpreted in relation to the geometric observations used by each estimator.

ElasticFusion [32] is a surfel-based dense RGB-D SLAM method that estimates camera pose through frame-to-model registration and fuses observations into a dense surface map. InfiniTAM [33] is a volumetric reconstruction framework that combines camera tracking with depth-map integration. These surfel-based and volumetric methods extend the trajectory comparison to include dense approaches alongside the point-feature-based ORB-SLAM3 baseline.

**Figure 3:**
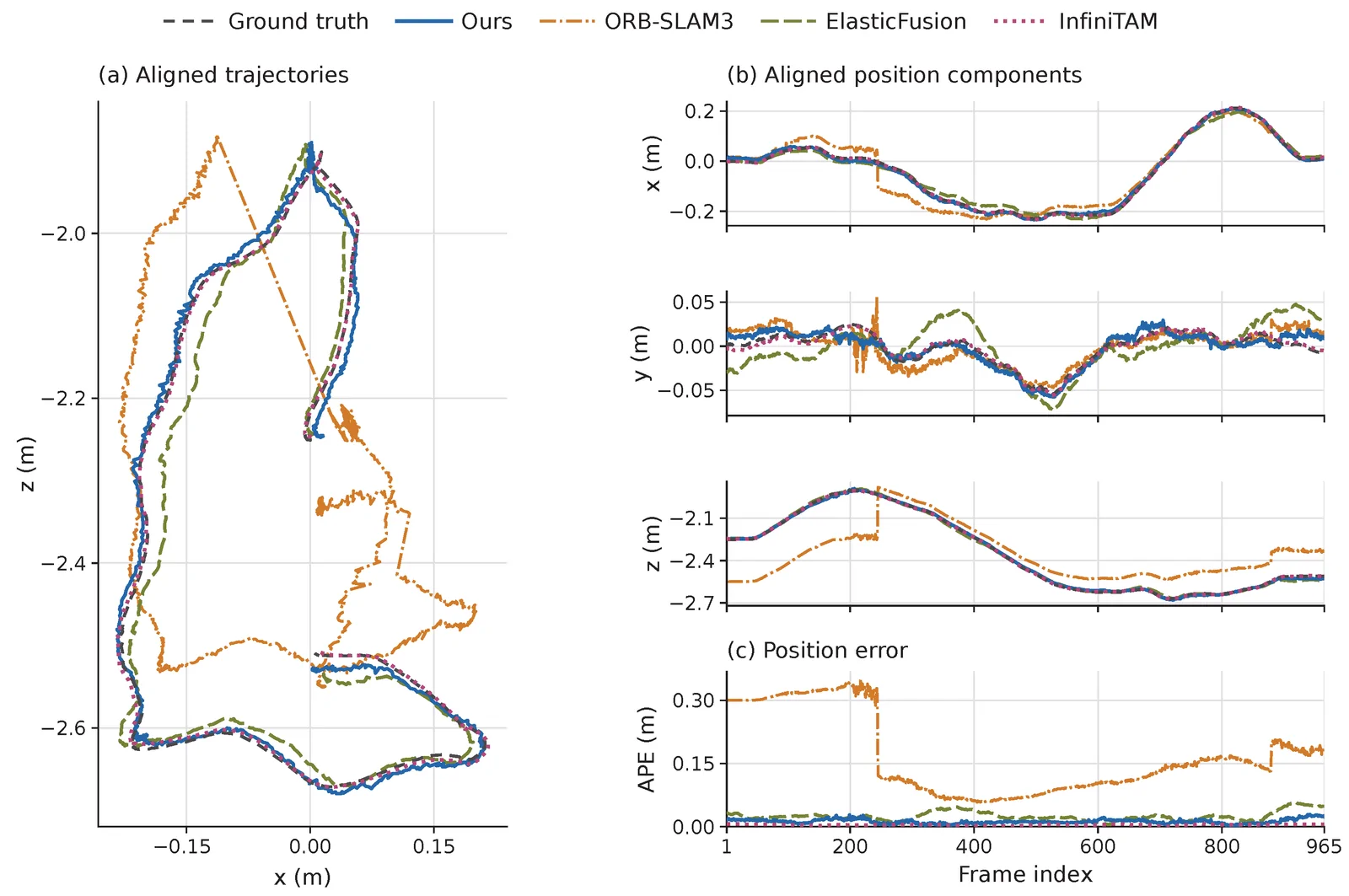
Trajectory comparison on ICL-NUIM lr_kt1. All four estimated trajectories are independently aligned to ground truth on 965 common valid frames using full-trajectory Sim(3) alignment. (**a**) Aligned trajectories projected onto the *x*–*z* plane. (**b**) Aligned position components. (**c**) Absolute position error (APE) in meters. ElasticFusion and InfiniTAM positions are from public SLAMBench run logs [31].

Figure 3 shows the trajectories and position errors of the proposed method, ORB-SLAM3, ElasticFusion, and InfiniTAM on lr_kt1. InfiniTAM is closest to ground truth in these records, with a position-error RMSE of approximately 0.0046 m; the corresponding values for the proposed method and ElasticFusion are 0.0135 m and 0.0273 m. The proposed method has lower overall position error than ElasticFusion and ORB-SLAM3, although ElasticFusion has lower error over some local intervals. These results show that the proposed method achieves good trajectory accuracy on this sequence, while InfiniTAM achieves higher accuracy in the compared record.

**Figure 4:**
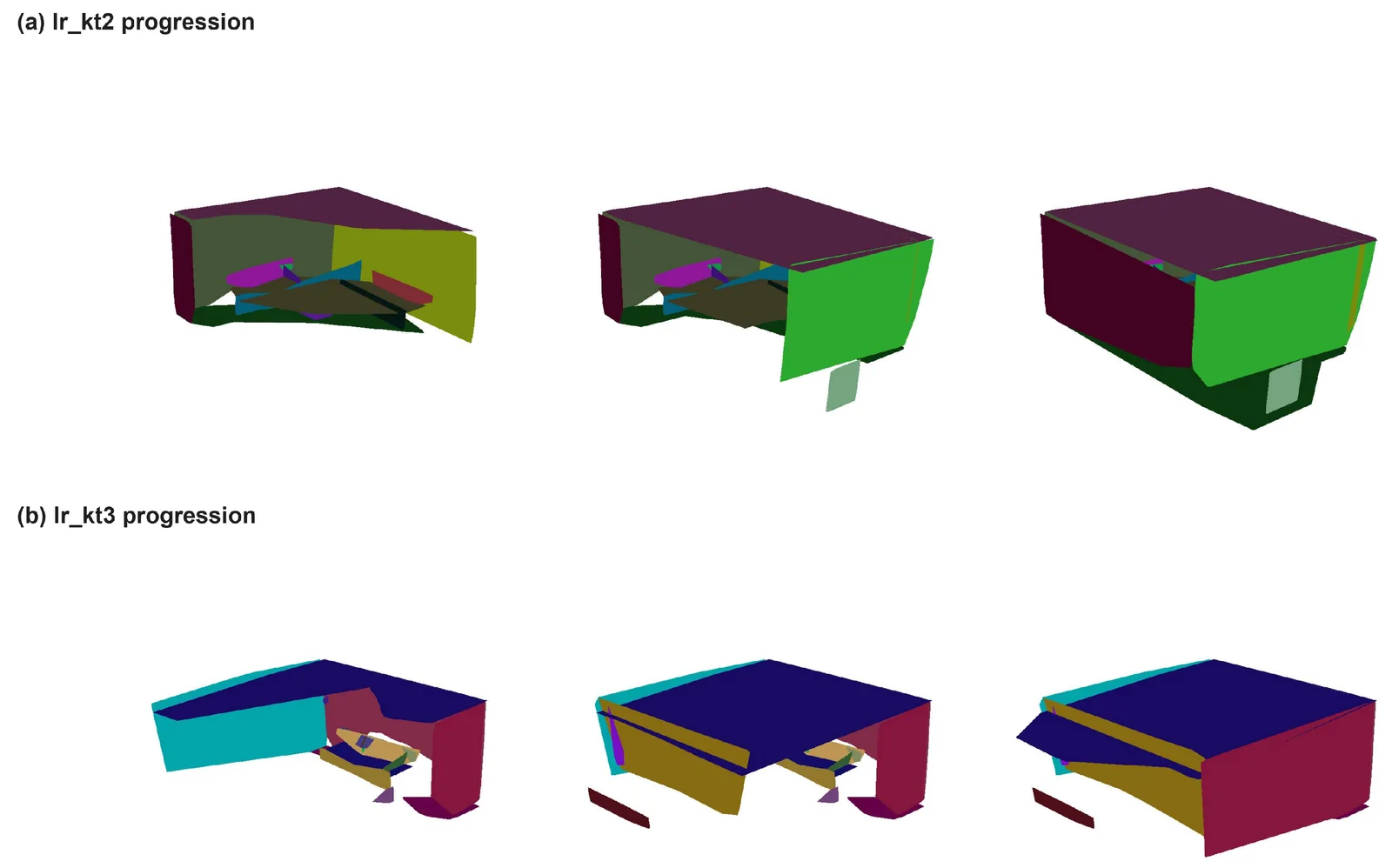
Plane-map progression for ICL-NUIM. (**a**) Three successive mapping stages from lr_kt2, shown from left to right. (**b**) Three successive stages from lr_kt3. Different colors distinguish persistent plane instances; the colors are identifiers rather than a quantitative scale. Plane support expands as accepted keyframes contribute associated observations.

Figure 4 shows early, intermediate, and later stages of the mapping process for two trajectories. Dominant walls and ceiling/floor surfaces grow as new keyframes are associated with persistent instances.

**Figure 5:**
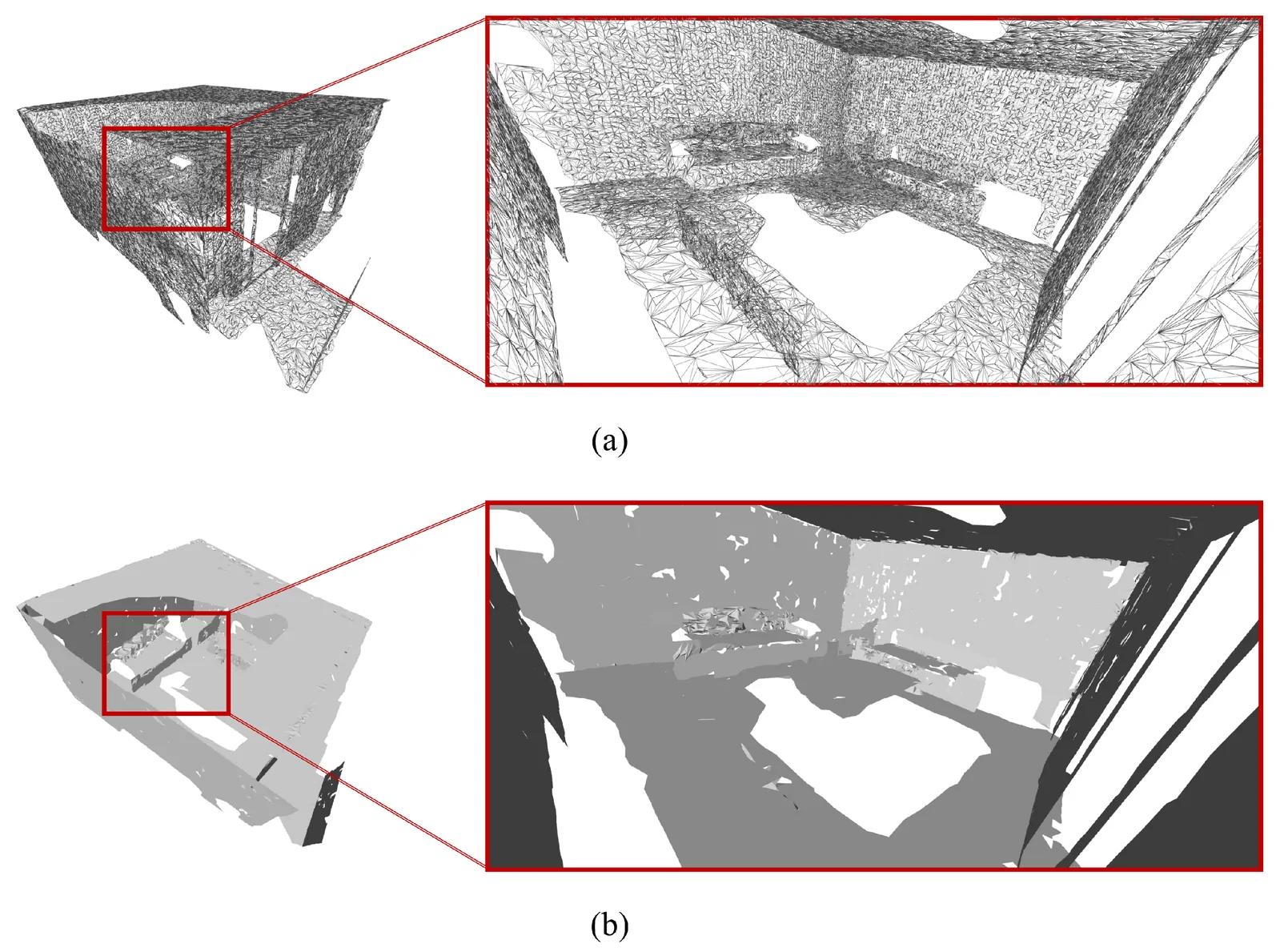
Surface-construction detail. (**a**) A wireframe view after plane-support fusion, voxel processing, projection, and greedy triangulation, with an enlarged region showing a surface gap. (**b**) The corresponding surface after filling gaps supported by the surrounding planar structure. Red boxes and connecting lines identify the enlarged regions.

Figure 5 shows the plane-instance representation at two stages of surface construction. Triangulation exposes fine geometry and missing regions, while plane-supported gap filling produces a more continuous surface for visualization.

**Figure 6:**
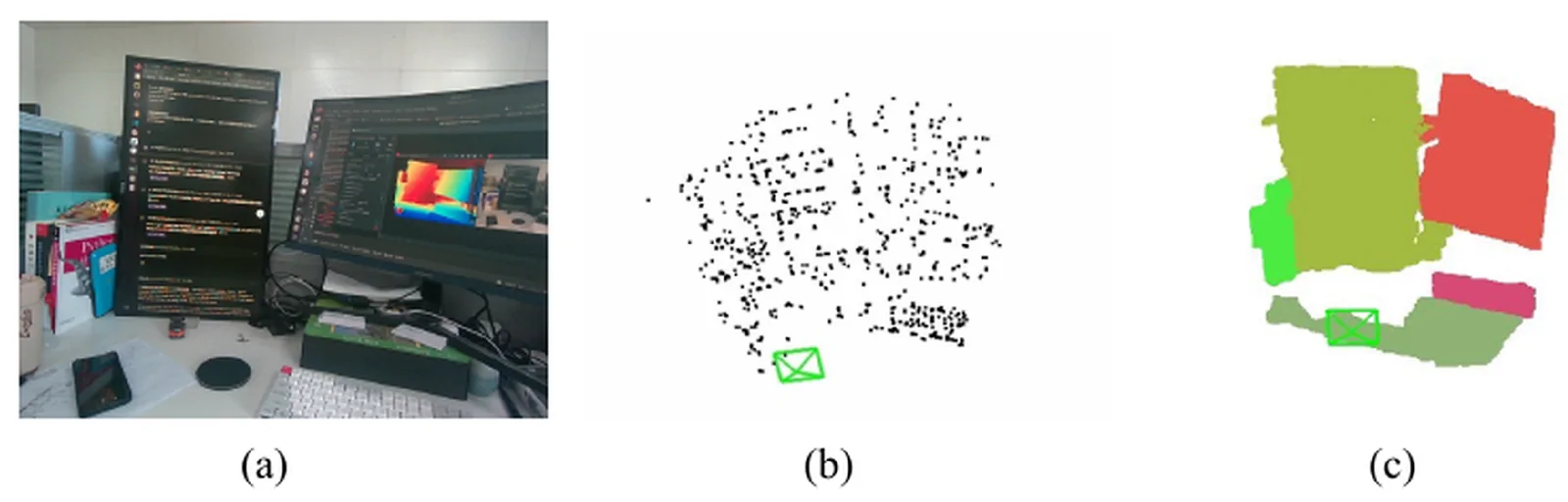
Indoor plane-instance representation. (**a**) RGB reference. (**b**) Sparse point landmarks, shown in black, and the camera-pose wireframe, shown in green. (**c**) Fused plane-instance mesh, with different surface colors distinguishing plane instances and the green wireframe indicating the camera pose. Surface colors are instance identifiers rather than an error or semantic-class scale.

Figure 6 shows the RGB reference, sparse landmarks, and fused plane-instance mesh for the persistent structural representation. Table 8 separately quantifies the final surface generated by this mapping process. The proposed map has RMS values of 0.034, 0.052, 0.055, and 0.020 m. KinectFusion has lower RMS on lr_kt0–2, while the proposed method is lowest on lr_kt3. The proposed cross-sequence range is 0.035 m, compared with 0.087 m for DVO-SLAM, 0.142 m for KinectFusion, and 0.136 m for RGB-D SLAM. This range describes variation across the four sequences. KinectFusion integrates dense depth observations into a volumetric surface, while the proposed method constructs a mesh from fused plane support. Planar meshing emphasizes dominant structural surfaces and approximates local departures from planarity; this representation difference is relevant when interpreting surface RMS across scenes.

**Figure 7:**
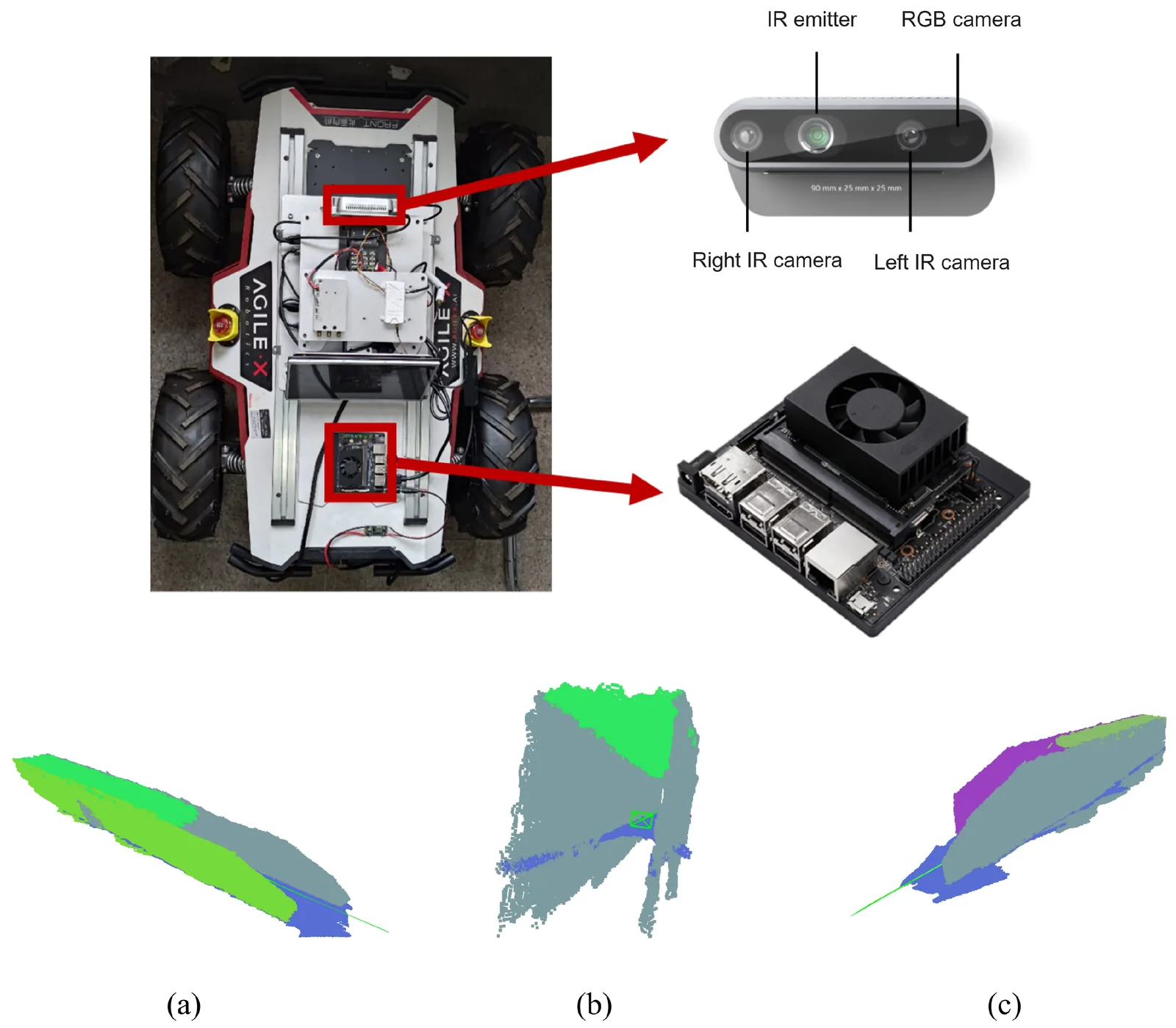
Onboard RGB-D mapping demonstration. The upper panel shows the mobile robot and close-up views of the Intel RealSense D435i and NVIDIA Jetson Xavier NX (8 GB); red boxes and arrows link the installed devices to their close-up views. The lower panels show the approximately 40 m corridor plane map: (**a**) an oblique overview; (**b**) a view showing the green camera-pose wireframe; (**c**) another oblique view. The map was obtained using RGB-D sensing alone at 640×480 and a configured rate of 15 frames/s. Different surface colors distinguish plane instances.

The views in Figure 7 recover the long planar corridor structure from several camera viewpoints. This onboard result demonstrates operation of the complete RGB-D sensing, tracking, and persistent plane-mapping path in a physical structured indoor environment.

The compact indoor vocabulary provides the persistent visual counterpart to the plane instances.

Table 9 reports its model storage, loading, and database-query costs. The indoor vocabulary occupies 15.7 MB compared with 145.3 MB for the general model, an 89.2% reduction. Loading time decreases from 3235 to 255 ms (92.1%), and one-query latency decreases from 0.10 to 0.02 ms (80.0%).

### 5.3. Accuracy–Resource Trade-Offs and Observed Process Demand

The trajectory gains in Table 4 were accompanied by higher tracking cost. Median tracking-call latency increased from 16.174 ms for P to 50.589 ms for P + L and 92.505 ms for P + L + Π. Read with the corresponding ATE RMSE values of 0.052350, 0.021353, and 0.017041 m, these configuration-level measurements define the observed accuracy–latency trade-off.

The following detector timings and process-resource measurements complement the tracking latency and vocabulary costs reported above.

#### 5.3.1. Detector-Level Trade-Off

Table 10 reports detector timing and corner counts. On texture_far, AGAST 5–8 required 0.534 ms compared with 0.648 ms for FAST, a 17.6% reduction. On texture_near, the corresponding values were 0.432 and 0.500 ms, a 13.6% reduction. Their arithmetic mean is 15.6%. The same configuration returned 221 versus 550 corners on texture_far and 184 versus 443 on texture_near. The fixed 5–8 mask therefore offers a detector-level speed/candidate-density trade-off in textured scenes.

The no-texture rows show scarce point support. AGAST 5–8 had higher latency than FAST on both sequences and returned averages of 0 and 2 corners per frame; FAST returned averages of 8 and 4. These counts show scarce point support for both detectors, while extended boundaries and depth surfaces remain available to the hybrid tracker.

#### 5.3.2. Whole-Process Resource Profile

Table 11 reports the three-run P + L + Π process profile. Median RSS was 840.5 MiB and remained below 1.08 GiB at peak. Median CPU demand corresponded to approximately 1.84 logical cores. System CPU had a median of 21.6% and a maximum of 33.4%. Swap consumption remained zero during the 221 valid monitored samples.

### 5.4. Scene-Dependent Fixed-Weight Sensitivity

Table 12 shows scene-dependent sensitivity. On notexture_far, the reference has the lowest median ATE, 11.85 mm; 0.60/0.10/0.30 gives 15.56 mm, an increase of 3.71 mm (31.3%). On texture_near, 0.80/0.10/0.10 gives 10.09 mm versus 11.87 mm for the reference, a reduction of 1.78 mm (15.0%). For ICL lr_kt1, 0.60/0.30/0.10 gives 6.89 mm versus 7.95 mm for the reference. On ICL lr_kt3, the reference also has the lowest median ATE, 25.27 mm, compared with 26.95–29.01 mm for the other four settings. Associated-pose coverage is 99.50–99.93% across the 120 runs. The median and range summarize between-run variation for each sequence and configuration. Table 4 reports a single run for each modality configuration; Table 12 summarizes three runs for each fixed-weight configuration.

## 6. Discussion

### 6.1. Complementary Geometric Constraints in Low-Texture Scenes

Point, line, and plane observations provide complementary geometric constraints because they operate over different spatial supports. Point reprojection relates localized image observations to mapped landmarks. Endpoint-to-line residuals constrain projected endpoints relative to extended image boundaries, while direct-plane residuals describe surface orientation and distance along the plane normal. Parallel and perpendicular plane relations add orientation constraints between structural surfaces. In low-texture scenes, these measurement families can supply constraint directions that are weakly represented by the available point observations.

The progression from P to P + L and P + L + Π in Table 4 is consistent with this interpretation. Translational ATE RMSE decreased from 0.052350 to 0.021353 and 0.017041 m, and translational and rotational RPE followed the same ordering. The near-zero point counts in the no-texture detector tests further show that extended boundaries and depth planes provide geometric support beyond that available from either point detector.

The P + L + Π result is also competitive within the evaluated low-texture regime. Its 0.017041 m ATE RMSE was 67.4% below P, 20.2% below P + L, and 10.3–18.9% below the three selected published PLP results on the same TUM sequence. The system produced evaluable trajectories on both no-texture sequences, compared with 0/10 valid trajectories for each point-only baseline. Together, these observations connect complementary structural measurements with lower trajectory error and continued tracking availability under point-feature degradation.

### 6.2. Fixed Parameterization and Effective Measurement Influence

The reported estimator keeps its nominal information scales and modality coefficients fixed during each run. The P + L + Π reference uses coefficients of 0.70, 0.15, and 0.15; the sensitivity runs use the predefined alternatives in Table 12. Residual-specific information matrices retain their configured scales. This fixed parameterization makes each ablation mode a defined measurement configuration. The sensitivity results in Table 12 show that alternative fixed settings can yield lower ATE in particular scenes, while the reference is lowest on notexture_far and lr_kt3. The fixed setting provides a reproducible cross-sequence reference, and the evaluated grid characterizes scene-dependent variation.

Effective measurement contributions remain scene-dependent under fixed nominal coefficients. Association determines which observations enter the objective, Huber weighting limits large residuals, and fixed residual thresholds remove outliers between optimization rounds. The number, geometry, and residual magnitude of retained point, line, and plane observations therefore alter their realized influence on pose estimation while the configured coefficients remain constant.

### 6.3. Persistent Structural and Visual Representations

The plane instances and indoor vocabulary preserve two forms of state alongside frame-level estimation. Multiple observations of the same physical plane from frames selected for map insertion enlarge persistent plane support, after which meshing and plane-supported gap treatment form the structural surface. Across the four ICL-NUIM sequences, reconstruction RMS remained within 0.020–0.055 m, while the mapping progression illustrates how plane identities accumulate support over time. The approximately 40 m corridor map further confirms operation of the RGB-D sensing and persistent plane-mapping path on the robot platform.

The compact indoor vocabulary serves the visual place-recognition path. Relative to the evaluated general vocabulary, it reduced model storage from 145.3 to 15.7 MB, loading time from 3235 to 255 ms, and one-query latency from 0.10 to 0.02 ms. The geometric and visual representations are thus persistent for different purposes: one consolidates planar structure, while the other supports keyframe retrieval.

### 6.4. Accuracy, Computational Cost, and Observed Resource Use

The accuracy improvement is accompanied by higher tracking cost. The largest observed ATE reduction occurred from P to P + L (59.2%); P + L + Π provided a further 20.2% reduction relative to P + L. Median tracking-call latency increased from 16.174 ms for P to 50.589 ms for P + L and 92.505 ms for P + L + Π. The P + L + Π value is of the same order as the approximately 91 ms reported by PLPF-VSLAM [8]. At the point front end, fixed AGAST 5–8 reduced detector-only latency by an average of 15.6% on the two textured sequences while returning fewer candidates. These values characterize two computation layers: detector-only timing measures the point front end, and tracking-call latency measures the complete tracking call.

The process profile adds a whole-process view in the stated virtual-machine environment. Across 221 valid samples from three P + L + Π runs, median RSS was 840.5 MiB and peak RSS remained below 1.08 GiB. Median CPU demand was approximately 1.84 logical cores, median system CPU was 21.6%, and swap consumption remained zero. Read together with vocabulary storage and loading, these measurements distinguish per-call computation, vocabulary model cost, initialization cost, and sampled runtime demand.

### 6.5. Evaluation Context

TUM RGB-D evaluates tracking under point-feature degradation, and ICL-NUIM evaluates pose and surface reconstruction. The corridor experiment demonstrates the RGB-D sensing and plane-mapping pipeline on the D435i and Jetson Xavier NX platform. The reported detector timings and process-resource measurements apply to the Ubuntu 18.04 VirtualBox environment described in Section 4.

## 7. Conclusions

This paper examined complementary structural estimation and persistent representation in RGB-D SLAM for low-texture indoor environments. The system combines point-reprojection, endpoint-to-line, direct-plane, and structural plane-relation residuals under fixed nominal information scales and modality coefficients. It also maintains persistent plane instances for surface reconstruction and a compact indoor visual vocabulary for keyframe-level place recognition.

On TUM RGB-D fr3/structure_notexture_near, translational ATE RMSE decreased from 0.052350 m for P to 0.021353 m for P + L and 0.017041 m for P + L + Π, while median tracking-call latency increased from 16.174 to 50.589 and 92.505 ms. The P + L + Π ATE RMSE was 10.3–18.9% below the three selected published PLP results on the same sequence. Across four ICL-NUIM sequences, reconstruction RMS remained within 0.020–0.055 m. The compact vocabulary required 15.7 MB and loaded in 255 ms. Across three monitored P + L + Π runs, median RSS was 840.5 MiB, peak RSS remained below 1.08 GiB, median CPU demand was approximately 1.84 logical cores, and swap consumption remained zero.

Adding line and plane observations improved trajectory accuracy under weak point support at a higher tracking latency. The compact indoor vocabulary reduced storage and initialization costs, and the approximately 40 m corridor demonstration showed the integrated RGB-D sensing and plane-mapping pipeline on the robot platform. 

## Figures and Tables

**Table 1:** Execution schedule for sensing, optimization, and persistent updates.

Trigger	Operations	Persistent Effect
Every RGB-D frame	Point and line extraction from RGB; valid-depth filtering and plane extraction; association; fixed-information pose optimization	Updates the current pose and tracking state; no meshing or database insertion
Accepted keyframe	Coplanar plane association, instance fusion, voxel filtering, and meshing	Updates persistent plane instances and their structural surface support
Accepted keyframe	Descriptor quantization and keyframe-database query/insertion	Updates the BoW representation used for place-recognition candidates

**Table 2:** Fixed-information optimization and association configuration. Information scales, modality weights, and residual thresholds remain constant within each run.

Quantity	Value	Role and Source
Point information and Huber thresholds	Inverse scale-dependent pyramid variance; $\sqrt{5.991}$ for monocular and $\sqrt{7.815}$ for RGB-D/stereo projection	The pyramid level fixes the point residual scale. The two values are the conventional 95% chi-square thresholds for two- and three-dimensional point projections used in ORB-SLAM2 [1].
Fixed modality weights	Point/line/plane: 0.70/0.15/0.15	Reference configuration; the sensitivity analysis varies predefined configurations between runs (Section 4.5).
Line endpoint edges	One unary edge per 3-D endpoint; unit information on the active algebraic scalar embedded in $I_{3}$; Huber $\sqrt{7.815}$; rejection threshold 2×5.991	The Huber loss limits large endpoint residuals. If either endpoint exceeds the rejection threshold, the complete segment is rejected. These fixed implementation settings follow the PlanarSLAM framework [4].
Plane information $\Omega_{k}^{\pi }$	Angular scale $0.5^{\circ }$ and distance scale $50\;m^{-1}$; angular entries $3282.8/0.5^{2}$ and distance entry $50^{2}$	The backend converts the angular entries to inverse-radian-squared information; the distance entry corresponds to a 0.02 m scale. The values follow the dataset configuration of the PlanarSLAM framework [4].
Plane Huber threshold	$\sqrt{100}$	Limits the influence of large direct-plane residuals; used as a fixed implementation setting.
Parallel/perpendicular relation edges	Angular scale $0.1^{\circ }$; both diagonal information entries are $3282.8/0.1^{2}$; Huber $\sqrt{50}$; rejection threshold $\chi^{2}=50$	Each edge contains two angular components. The Huber loss and rejection threshold robustify the structural orientation relations.
Plane extraction	TUM: minimum 2000 support pixels and 0.05 m distance; ICL: minimum 500 pixels and 0.03 m distance	Minimum support suppresses small patches, and the distance threshold controls region growth. The dataset-specific values follow the corresponding baseline configurations.
Plane association/relations	Distance 0.05 m; normal dot product 0.985; parallel 0.9962; perpendicular absolute dot product 0.08716	These values correspond to approximately $10^{\circ }$ for plane association and $5^{\circ }$ deviations from parallel or perpendicular structure.
Robust optimization schedule	Four rounds of ten Levenberg–Marquardt iterations	Outliers are reclassified between rounds before the next optimization pass.

**Table 3:** Overview of evaluation datasets, metrics, and objectives.

Evaluation	Dataset and Metric	Objective
Detector trade-off	Four structured TUM RGB-D sequences; mean detector-only latency and corners/frame in an Ubuntu 18.04 VirtualBox 7.0 guest with 10 vCPUs and 8 GB RAM on a Windows 11 host with an Intel Core i7-12700K processor (Intel Corporation, Santa Clara, CA, USA)	Compare detector speed and candidate density under common hardware and inputs
Modality ablation	TUM RGB-D fr3/structure_ notexture_near; translational ATE RMSE, translational and rotational one-step RPE, trajectory coverage, and tracking-call latency	Quantify the effect of adding line and plane measurements to point tracking
Weak-texture availability	Two structured TUM RGB-D no-texture sequences; valid trajectories over ten runs for the point-only baselines	Assess trajectory availability when point support is scarce
PLP comparison	Published ATE RMSE values on fr3/structure_notexture_near	Compare P + L + Π accuracy with closely related published methods
Cross-trajectory pose	ICL-NUIM lr_kt0–3; Sim(3)-aligned full-transformation RMSE index	Compare pose estimation across synthetic indoor trajectories
Structural mapping	ICL-NUIM lr_kt0–3; reconstruction RMS in meters	Quantify final-surface error and structural consistency across trajectories
Onboard system validation	D435i (Intel Corporation, Santa Clara, CA, USA), Jetson Xavier NX, 8 GB (NVIDIA Corporation, Santa Clara, CA, USA), and an approximately 40 m corridor; plane-map views	Validate integrated RGB-D sensing and persistent plane mapping on the robot platform
Process-resource profile	Three P + L + Π runs; 221 valid samples at an approximately 2 s interval; RSS, process CPU, system CPU, and swap	Quantify the observed runtime resource envelope
Place-recognition cost	General and compact indoor vocabularies; storage, loading, and one-query timing	Quantify storage, loading, and query reductions from the compact indoor vocabulary

**Table 4:** Modality-ablation performance on TUM RGB-D fr3/structure_notexture_near. ATE is translational RMSE after rigid SE(3) alignment with fixed scale and a 0.02 s association tolerance. RPE uses adjacent associated poses. Tracking denotes median tracking-call latency.

Mode	ATE (m)	${\mathrm{RPE}}_{t}$ (m/Step)	${\mathrm{RPE}}_{R}$ (deg/Step)	Tracking (ms)	Est./Assoc.
P	0.052350	0.016474	0.905	**16.174**	1051/1049
P + L	0.021353	0.008259	0.512	50.589	1054/1052
P + L + Π	**0.017041**	**0.007064**	**0.447**	92.505	1054/1052

Bold values indicate the lowest ATE, translational RPE, rotational RPE, and median tracking-call latency among the compared configurations.

**Table 5:** Trajectory availability on the two structured no-texture TUM RGB-D sequences. “0/10” denotes zero valid trajectories in ten runs; “valid” denotes an evaluable trajectory from the proposed system.

Sequence	ORB-SLAM2	ORB-SLAM3	Ours
notexture_far	0/10	0/10	Valid
notexture_near	0/10	0/10	Valid

**Table 6:** Published ATE RMSE results for closely related point–line–plane RGB-D SLAM methods on TUM RGB-D fr3/structure_notexture_near (lower is better). The UPLP-SLAM value is reproduced from Table 2 in Wang et al. [9]; the other values are taken from the cited primary sources. This table was compiled by the authors from these sources.

Method	Fusion	ATE RMSE (m)	Reduction by Ours
PLPF-VSLAM [8]	Adaptive PLP	0.0190	10.3%
PLP-SLAM [9]	Degeneracy-aware PLP	0.0200	14.8%
UPLP-SLAM [20]	Unified PLP	0.0210	18.9%
Ours	Fixed-weight PLP	**0.0170**	–

Bold indicates the lowest ATE RMSE among the compared methods.

**Table 7:** Pose-estimation comparison on ICL-NUIM using the unitless RMSE of the full-transformation error after Sim(3) alignment (lower is better). This metric is reported separately from the SE(3)-aligned ATE and RPE evaluation on TUM RGB-D. Bold marks the lowest value in each row.

Sequence	ORB-SLAM2	ORB-SLAM3	SP-SLAM	ManhattanSLAM	Ours
lr_kt0	0.021651	0.022303	**0.012408**	0.018964	0.021773
lr_kt1	0.383051	0.286710	0.286297	0.041794	**0.026922**
lr_kt2	0.035076	0.025236	**0.023659**	0.034908	0.024739
lr_kt3	0.015327	0.020544	0.114001	**0.015059**	0.019523

**Table 8:** Reported ICL-NUIM reconstruction RMS in meters (lower is better). Bold marks the lowest value in each row.

Sequence	DVO-SLAM	KinectFusion	RGB-D SLAM	Ours
lr_kt0	0.032	**0.011**	0.044	0.034
lr_kt1	0.061	**0.008**	0.032	0.052
lr_kt2	0.119	**0.009**	0.031	0.055
lr_kt3	0.053	0.150	0.167	**0.020**
Cross-sequence range	0.087	0.142	0.136	**0.035**

For the cross-sequence range, bold indicates the smallest range across the four sequences.

**Table 9:** Storage, loading time, and one-query latency for the general and compact indoor vocabularies.

Metric	General	Indoor	Reduction
Storage (MB)	145.3	15.7	89.2%
Loading time (ms)	3235	255	92.1%
One-query latency (ms)	0.10	0.02	80.0%

**Table 10:** Detector-only latency and returned corner count for five segment-test detector configurations. Measurements were obtained in an Ubuntu 18.04 VirtualBox 7.0 guest with 10 vCPUs and 8 GB RAM on a Windows 11 host equipped with an Intel Core i7-12700K processor and 16 GB physical memory. The fixed AGAST 5–8 configuration used by the proposed front end and the FAST reference are highlighted in bold.

Measure	Sequence	AGAST 5–8	AGAST 7–12s	AGAST 7–12d	OAST 9–16	FAST
Latency (ms)	notexture_far	**0.219**	0.221	0.222	0.434	**0.084**
	notexture_near	**0.210**	0.210	0.211	0.424	**0.076**
	texture_far	**0.534**	0.503	0.577	0.901	**0.648**
	texture_near	**0.432**	0.404	0.453	0.742	**0.500**
Corners/frame	notexture_far	**0**	1	1	4	**8**
	notexture_near	**2**	1	2	9	**4**
	texture_far	**221**	438	473	564	**550**
	texture_near	**184**	347	388	474	**443**

**Table 11:** Runtime resource profile of the P + L + Π process across three monitored runs.

Metric	Median	P95	Maximum
Resident set size (MiB)	840.5	1010.2	1070.7
SLAM-process CPU (%)	183.8	222.9	228.6
System CPU (%)	21.6	27.6	33.4

Statistics pool 221 simultaneous-valid samples from three P + L + Π runs (79, 77, and 65 samples) recorded at an approximately 2 s interval. Under the Linux process convention, 100% process CPU corresponds approximately to one logical core. No swap consumption was observed in any valid sample.

**Table 12:** Fixed-weight sensitivity: translational ATE RMSE in millimeters after fixed-scale SE(3) alignment. Each cell is the median [minimum, maximum] of three runs (3/3 for every cell). Weights are point/line/plane; 0.70/0.15/0.15 is the reference. TUM labels abbreviate fr3/structure_; ICL uses lr_kt0–3.

Sequence	0.34/0.33/0.33	0.60/0.10/0.30	0.60/0.30/0.10	0.70/0.15/0.15	0.80/0.10/0.10
notexture_far	12.36 [11.97, 12.75]	15.56 [11.73, 15.61]	12.60 [11.36, 12.95]	11.85 [11.68, 12.18]	13.07 [12.84, 13.47]
notexture_near	16.68 [13.51, 20.03]	16.98 [14.32, 17.18]	18.33 [12.17, 19.73]	17.03 [15.56, 18.87]	17.75 [17.57, 20.04]
texture_far	16.53 [16.37, 18.00]	15.26 [14.04, 17.69]	15.32 [14.02, 15.46]	15.91 [15.57, 16.84]	15.58 [15.39, 15.85]
texture_near	13.28 [10.23, 13.55]	12.19 [11.91, 13.13]	14.32 [13.76, 14.50]	11.87 [9.82, 14.05]	10.09 [9.74, 12.68]
lr_kt0	8.24 [7.97, 8.79]	8.47 [8.33, 8.59]	7.74 [7.65, 8.07]	8.07 [7.63, 8.40]	8.19 [8.12, 8.21]
lr_kt1	7.33 [7.23, 7.37]	7.42 [7.03, 7.88]	6.89 [6.70, 7.79]	7.95 [7.80, 7.99]	7.48 [7.40, 7.64]
lr_kt2	10.67 [10.20, 12.04]	12.26 [12.15, 12.63]	11.54 [10.94, 13.69]	12.40 [11.57, 12.63]	12.12 [11.32, 12.52]
lr_kt3	28.13 [27.42, 30.98]	29.01 [26.69, 29.93]	28.04 [27.96, 30.73]	25.27 [25.17, 27.19]	26.95 [26.89, 27.23]

## Data Availability

The TUM RGB-D and ICL-NUIM datasets are publicly available from the benchmark resources cited in Section 4.

## Abbreviations

The following abbreviations are used in this manuscript:

APE	absolute pose error
ATE	absolute trajectory error
BoW	bag of words
LM	Levenberg–Marquardt
RGB-D	red–green–blue plus depth
RPE	relative pose error
RSS	resident set size
RMS	root mean square
SE(3)	special Euclidean group in three dimensions
SLAM	simultaneous localization and mapping

## References

[1] Mur-Artal R., Tardos J.D. (2017). ORB-SLAM2: An Open-Source SLAM System for Monocular, Stereo, and RGB-D Cameras. IEEE Trans. Robot..

[2] Campos C., Elvira R., Rodriguez J.J.G., Montiel J.M.M., Tardos J.D. (2021). ORB-SLAM3: An Accurate Open-Source Library for Visual, Visual-Inertial, and Multimap SLAM. IEEE Trans. Robot..

[3] Pumarola A., Vakhitov A., Agudo A., Sanfeliu A., Moreno-Noguer F. PL-SLAM: Real-Time Monocular Visual SLAM with Points and Lines. Proceedings of the IEEE International Conference on Robotics and Automation.

[4] Li Y., Yunus R., Brasch N., Navab N., Tombari F. RGB-D SLAM with Structural Regularities. Proceedings of the IEEE International Conference on Robotics and Automation.

[5] Yunus R., Li Y., Tombari F. ManhattanSLAM: Robust Planar Tracking and Mapping Leveraging Mixture of Manhattan Frames. Proceedings of the IEEE International Conference on Robotics and Automation.

[6] Cho H.M., Jo H.G., Kim E. (2022). SP-SLAM: Surfel-Point Simultaneous Localization and Mapping. IEEE/ASME Trans. Mechatron..

[7] Shu F., Wang J., Pagani A., Stricker D. Structure PLP-SLAM: Efficient Sparse Mapping and Localization Using Point, Line and Plane for Monocular, RGB-D and Stereo Cameras. Proceedings of the IEEE International Conference on Robotics and Automation.

[8] Yan J., Zheng Y., Yang J., Mihaylova L., Yuan W., Gu F. (2024). PLPF-VSLAM: An Indoor Visual SLAM with Adaptive Fusion of Point–Line–Plane Features. J. Field Robot..

[9] Wang X., Bi J., Yang S. (2024). Multi-Feature Fusion RGB-D SLAM Method for Indoor Weak-Texture Scenes. Navig. Position. Timing.

[10] Yan Y., Li J., Liu Q., Lv S., Wu Q. (2026). RGB-D SLAM: A Review of Methods and Performance Trade-Offs for Different Requirements. Sensors.

[11] Mair E., Hager G.D., Burschka D., Suppa M., Hirzinger G. (2010). Adaptive and Generic Corner Detection Based on the Accelerated Segment Test. Proceedings of the Computer Vision—ECCV 2010.

[12] Akinlar C., Topal C. (2011). EDLines: A Real-Time Line Segment Detector with a False Detection Control. Pattern Recognit. Lett..

[13] Feng C., Taguchi Y., Kamat V.R. Fast Plane Extraction in Organized Point Clouds Using Agglomerative Hierarchical Clustering. Proceedings of the IEEE International Conference on Robotics and Automation.

[14] Sturm J., Engelhard N., Endres F., Burgard W., Cremers D. A Benchmark for the Evaluation of RGB-D SLAM Systems. Proceedings of the IEEE/RSJ International Conference on Intelligent Robots and Systems.

[15] Handa A., Whelan T., McDonald J., Davison A.J. A Benchmark for RGB-D Visual Odometry, 3D Reconstruction and SLAM. Proceedings of the IEEE International Conference on Robotics and Automation.

[16] Rublee E., Rabaud V., Konolige K., Bradski G. ORB: An Efficient Alternative to SIFT or SURF. Proceedings of the IEEE International Conference on Computer Vision.

[17] Zou Y., Eldemiry A., Li Y., Chen W. (2020). Robust RGB-D SLAM Using Point and Line Features for Low Textured Scene. Sensors.

[18] Wang R., Di K., Wan W., Wang Y. (2018). Improved Point-Line Feature Based Visual SLAM Method for Indoor Scenes. Sensors.

[19] Xia Y., Wu X., Ma T., Zhu L., Cheng J., Zhu J. (2024). Multi-Robot Collaborative Mapping with Integrated Point-Line Features for Visual SLAM. Sensors.

[20] Yang H., Yuan J., Gao Y., Sun X., Zhang X. (2023). UPLP-SLAM: Unified Point–Line–Plane Feature Fusion for RGB-D Visual SLAM. Inf. Fusion.

[21] Yang G., Liu P., Ma W. (2025). Enhanced RGB-D SLAM through Orthogonal Plane Constraints and Point–Line–Plane Collaborative Optimization. PLoS ONE.

[22] Guo R., Peng K., Fan W., Zhai Y., Liu Y. (2019). RGB-D SLAM Using Point–Plane Constraints for Indoor Environments. Sensors.

[23] Newcombe R.A., Izadi S., Hilliges O., Molyneaux D., Kim D., Davison A.J., Kohli P., Shotton J., Hodges S., Fitzgibbon A. KinectFusion: Real-Time Dense Surface Mapping and Tracking. Proceedings of the IEEE International Symposium on Mixed and Augmented Reality.

[24] Kerl C., Sturm J., Cremers D. Dense Visual SLAM for RGB-D Cameras. Proceedings of the IEEE/RSJ International Conference on Intelligent Robots and Systems.

[25] Endres F., Hess J., Sturm J., Cremers D., Burgard W. (2014). 3-D Mapping With an RGB-D Camera. IEEE Trans. Robot..

[26] Keetha N., Karhade J., Jatavallabhula K.M., Yang G., Scherer S., Ramanan D., Luiten J. SplaTAM: Splat, Track & Map 3D Gaussians for Dense RGB-D SLAM. Proceedings of the IEEE/CVF Conference on Computer Vision and Pattern Recognition.

[27] Zhang J., Ganesh P., Volle K., Willis A., Brink K. (2021). Low-Bandwidth and Compute-Bound RGB-D Planar Semantic SLAM. Sensors.

[28] Marton Z.C., Rusu R.B., Beetz M. On Fast Surface Reconstruction Methods for Large and Noisy Point Clouds. Proceedings of the IEEE International Conference on Robotics and Automation.

[29] Galvez-Lopez D., Tardos J.D. (2012). Bags of Binary Words for Fast Place Recognition in Image Sequences. IEEE Trans. Robot..

[30] Huber P.J., Ronchetti E.M. (2009). Robust Statistics.

[31] PAMELA Project (2018). SLAMBench Public ICRA 2018 Results: ElasticFusion and InfiniTAM Run Logs for ICL-NUIM Living Room Trajectory 1. https://github.com/pamela-project/slambench/tree/8a9deeaa5124e824fa7f8e16eea97ad0bd0404c6/icra2018_results/1080.

[32] Whelan T., Leutenegger S., Salas Moreno R., Glocker B., Davison A. ElasticFusion: Dense SLAM Without a Pose Graph. Proceedings of the Robotics: Science and Systems.

[33] Prisacariu V.A., Kähler O., Cheng M.M., Ren C.Y., Valentin J., Torr P.H.S., Reid I.D., Murray D.W. (2014). A Framework for the Volumetric Integration of Depth Images. arXiv.

